# De‐Escalating Surgery in Merkel Cell Carcinoma With Clinical Nodal Disease

**DOI:** 10.1002/jso.28037

**Published:** 2024-12-15

**Authors:** Neha Shafique, Emily Ertmann, Gabriella N. Tortorello, Cimarron E. Sharon, Giorgos C. Karakousis, John T. Miura

**Affiliations:** ^1^ Department of Surgery Hospital of the University of Pennsylvania Philadelphia Pennsylvania USA; ^2^ Department of Surgery, Division of Endocrine and Oncologic Surgery Hospital of the University of Pennsylvania Philadelphia Pennsylvania USA

**Keywords:** de‐escalating surgery, Merkel cell carcinoma, nodal surgery, single node excision

## Abstract

**Background:**

Merkel cell carcinoma (MCC) is a radiosensitive aggressive skin cancer that spreads via the lymphatics. There is uncertainty regarding the optimal management of the nodal basin for patients with MCC with clinically positive nodes. We study the efficacy of single node excision (SNE) as an alternative to a therapeutic lymph node dissection (TLND) in patients with limited nodal disease.

**Methods:**

We performed a retrospective cohort study of patients with MCC with clinical nodal disease who underwent resection in the form of either SNE or TLND using the National Cancer Database. The association between type of surgery and overall survival (OS) was estimated using the Kaplan–Meier method and Cox proportional hazards modeling.

**Results:**

1835 patients met all inclusion criteria with 281 patients undergoing SNE and the remainder undergoing TLND. Patients receiving SNE and TLND were equally likely to receive radiation to the nodal basin (SNE 44.5% vs. TLND 48.5%, *p* = 0.22). There was no difference in 5‐year OS between patients who received SNE versus TLND (SNE 43.9% vs. TLND 44.7%, *p* = 0.36). This persisted in a multivariable Cox proportional hazards model in which receipt of SNE remained not significantly associated with survival after adjusting for clinical and treatment factors including receipt of radiation (Hazard Ratio [HR] 1.17, 95% CI 0.96–1.42, *p* = 0.11). In patients undergoing SNE with radiation, 5‐year OS was 54.4% (95% CI 44.1–63.6).

**Conclusions:**

TLND is not associated with a survival advantage over SNE. Further prospective study into patterns of recurrence and safety of SNE is needed.

## 1 Introduction

Merkel cell carcinoma (MCC) is a rare and aggressive cutaneous neuroendocrine malignancy with high rates of recurrence and mortality [1, 2]. MCC demonstrates a propensity to metastasize, particularly, to regional lymph nodes, underscoring the importance of nodal evaluation at the time of local excision [3]. When nodes are found to be positive for microscopic or macroscopic disease, several options are considered for management. Given the rarity of MCC, clinical guidelines and practice often apply findings from melanoma, a far more common skin cancer that also spreads via lymphatics.

Practice patterns in the treatment of melanoma with micrometastases confirmed through sentinel lymph node biopsy (SLNB) have shifted away from completion lymph node dissection (CLND) following the publication of Multicenter Selective Lymphadenectomy Trial‐II (MSLT‐II) [4, 5]. For patients with a single clinically evident metastatic melanoma node (cN1b), however, the surgical recommendation remains therapeutic lymph node dissection (TLND). A previous study on patients with melanoma with cN1b disease suggests that TLND may be safely avoided in select patients without impacting survival in favor of more limited removal of the clinically positive node (single node excision [SNE]) [6].

Currently, for MCC patients with clinically positive nodes, National Comprehensive Cancer Network (NCCN) guidelines recommend treatment with TLND and/or radiation to the nodal basin [7]. SNE may provide another therapeutic option for patients with limited clinical nodal disease which can be surgically excised, yet avoid some of the morbidity or wound complications associated with a TLND. This may allow for the timely delivery of adjuvant radiation to address residual microscopic or macroscopic disease. Using a national cohort, we sought to determine differences in survival outcomes between MCC patients with cN1b clinical nodal disease undergoing TLND or SNE.

## 2 Methods

We conducted a retrospective cohort study using the American College of Surgeons Commission on Cancer (CoC) National Cancer Database (NCDB) which collects data from over 1500 CoC‐affiliated referring facilities [8]. Patients with MCC with a known primary and clinical nodal disease and no evidence of distant metastases (American Joint Committee on Cancer 8th edition cN1bM0) who underwent surgical resection in the form of either a TLND or SNE were identified between the years of 2004 and 2020. Patients with unknown lymph node dissection and unknown nodal basin radiation status were excluded from analysis. Univariate analysis of clinicopathologic factors was performed using *χ* ^2^ tests for categorical variables and rank‐sum tests for continuous variables. A multivariable analysis investigating factors associated with receipt of TLND was performed utilizing variables found to be significant on univariate analysis (*p* value < 0.05). The association between type of surgery performed on 5‐year overall survival (OS) was estimated using the Kaplan–Meier method and Cox proportional hazards modeling with subgroup analyses for receipt of nodal basin radiation and rates of nodal positivity in TLND. Statistical analyses were performed using Stata Version 17 (StataCorp LLC). This study was exempt from Institutional Board Review approval.

## 3 Results

Of the 1835 patients included, the median age was 73 years, and most were male (*n* = 1292, 70.4%) and White (*n* = 1766, 96.2%). Most patients underwent TLND (*n* = 1554, 84.7%) with the remainder undergoing a more limited SNE.

There was no difference in many baseline characteristics between the two groups including sex, race, and comorbidities (Table 1). Patients undergoing TLND had a median number of 17 nodes examined with a median of three nodes with pathologically proven disease. Patients receiving SNE and TLND were equally likely to receive radiation to the nodal basin (SNE 44.5% vs. TLND 48.5%, *p* = 0.22). On multivariable analysis, older patients were less likely to undergo TLND (increasing age odds ratio [OR] 0.98, 95% confidence interval [CI] 0.96–0.99, *p* = 0.03). Patients who underwent TLND were also more likely to have a head and neck primary tumor (OR 1.70, 95% CI 1.07–‐2.71, *p* = 0.02) and receive treatment at an academic center (OR 1.75, 95% CI 1.32–2.31, *p* < 0.001).

**Table 1:** Clinicopathologic factors of Merkel cell carcinoma patients with clinical nodal disease who underwent single node excision (SNE), or therapeutic lymph node dissection (TLND) as identified from the National Cancer Database (2004–2020).

	SNE (*n* = 281)	TLND (*n* = 1554)	*p*‐value
Number of nodes examined (median; IQR)	1 (1–1)	17 (8–25)	< 0.01
Number of positive nodes (median; IQR)	1 (1–1)	3 (1–5)	< 0.01
Age at diagnosis, median (interquartile range; IQR)	75 (66–82)	73 (64–80)	0.01
Sex, *n* (%)			
Male	184 (65.5)	1108 (71.3)	
Female	97 (34.5)	446 (28.7)	0.05
Race, *n* (%)			
White	270 (96.1)	1496 (96.3)	
Black	5 (1.8)	25 (1.6)	
Asian American Pacific Islander	4 (1.4)	19 (1.2)	
Unknown	2 (0.7)	14 (0.9)	0.97
Ethnicity, *n* (%)			
Non‐Hispanic	263 (93.6)	1452 (93.4)	
Hispanic	7 (2.5)	55 (3.5)	
Not reported	11 (3.9)	47 (3.0)	0.50
Insurance status, *n* (%)			
Uninsured	3 (1.1)	6 (0.4)	
Private insurance	60 (21.4)	426 (27.4)	
Medicaid	5 (1.8)	32 (2.1)	
Medicare	210 (74.7)	1041 (67.0)	
Unknown	3 (1.1)	49 (3.2)	0.02
Charlson‐Deyo Score, *n* (%)			
0	204 (72.6)	1071 (68.9)	
1	53 (18.9)	309 (19.9)	
2	13 (4.6)	97 (6.2)	
3	11 (3.9)	77 (5.0)	0.54
Site of primary tumor, *n* (%)			
Head/neck	62 (22.1)	522 (33.6)	
Trunk	34 (12.1)	174 (11.2)	
Extremity	89 (31.7)	486 (31.3)	
Skin, not otherwise specified	96 (34.2)	372 (23.9)	< 0.01
T‐stage, *n* (%)			
T0	65 (23.1)	244 (15.7)	
T1	67 (23.8)	432 (27.8)	
T2	43 (15.3)	369 (23.7)	
T3	17 (6.0)	103 (6.6)	
T4	5 (1.8)	43 (2.8)	
Unknown T‐stage	84 (29.9)	363 (23.4)	< 0.01
Region, *n* (%)			
Northeast	84 (29.9)	354 (22.8)	
South	96 (34.2)	549 (35.3)	
Midwest	51 (18.1)	395 (25.4)	
West	49 (17.4)	248 (16.0)	
Unknown	1 (0.4)	8 (0.5)	0.03
Urban designation, *n* (%)			
Metropolitan	234 (83.3)	1213 (78.1)	
Urban	31 (11.0)	232 (14.9)	
Rural	5 (1.8)	27 (1.7)	
Unknown	11 (3.9)	82 (5.3)	0.24
Facility academic status, *n* (%)			
Nonacademic	176 (62.6)	760 (48.9)	
Academic	104 (37.0)	786 (50.6)	
Unknown	1 (0.4)	8 (0.5)	0.00
Nodal basin radiation, *n* (%)			
None	156 (55.5)	801 (51.5)	
Received nodal basin radiation	125 (44.5)	753 (48.5)	0.22
Immunotherapy, *n* (%)			
None	253 (90.0)	1417 (91.2)	
Received immunotherapy	28 (10.0)	137 (8.8)	0.54
Adjuvant systemic therapy, *n* (%)			
None	231 (82.2)	1283 (82.6)	
Received adjuvant therapy	50 (17.8)	271 (17.4)	0.89

**Figure 1:**
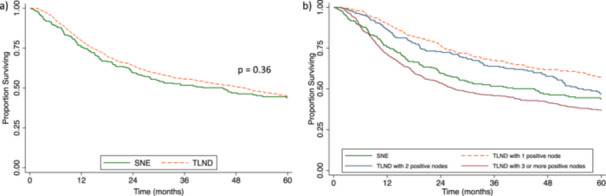
Kaplan–Meier curves of Merkel cell carcinoma patients with clinical nodal disease who underwent (a) single node excision (SNE) versus therapeutic lymph node dissection (TLND), and (b) SNE versus TLND with one positive node, two positive nodes, or three and more positive nodes.

In the overall cohort, there was no difference in 5‐year OS between patients who received SNE versus TLND (SNE 43.9% [95% CI 37.2–50.4] vs. TLND 44.7% [95% CI 41.8–47.5], *p* = 0.36) (Figure 1a). Receipt of an SNE remained not significantly associated with survival in the multivariable Cox proportional hazards model in the overall cohort (Hazard Ratio [HR] 1.17, 95% CI 0.96–1.42, *p* = 0.11) (Table 2).

**Table 2:** Cox proportional regression analysis for factors significantly associated with overall survival among patients with Merkel cell carcinoma patients with clinical nodal disease.

Factor	Hazard ratio (95% confidence interval)	*p*‐value
Type of surgery		
TLND	1.00 (reference)	
SNE	1.17 (0.96–1.42)	0.11
Nodal basin radiation		
None	1.00 (reference)	
Received nodal basin radiation	0.78 (0.68–0.91)	0.001
Age	1.04 (1.03–1.05)	< 0.001
Sex, *n* (%)		
Male	1.00 (reference)	
Female	0.80 (0.68–0.94)	0.007
Race, *n* (%)		
White	1.00 (reference)	
Black	1.14 (0.65–2.00)	0.65
Asian American Pacific Islander	0.45 (0.16–1.12)	0.11
Unknown	1.59 (0.74–3.43)	0.23
Ethnicity, *n* (%)		
Non‐Hispanic	1.00 (reference)	
Hispanic	0.89 (0.57–1.37)	0.59
Not reported	1.06 (0.75–1.49)	0.74
Insurance status, *n* (%)		
Private insurance	1.00 (reference)	
Uninsured	1.59 (0.59–4.33)	0.36
Medicaid	1.40 (0.77–2.53)	0.27
Medicare	0.93 (0.76–1.14)	0.47
Unknown	0.79 (0.50–1.24)	0.31
Charlson‐Deyo Score, *n* (%)		
0	1.00 (reference)	
1	1.33 (1.13–1.58)	0.001
2	1.56 (1.18–2.06)	0.002
3	2.42 (1.81–3.22)	< 0.001
Site of primary tumor, *n* (%)		
Trunk	1.00 (reference)	
Head/neck	1.15 (0.91–1.46)	0.23
Extremity	0.89 (0.71–1.13)	0.34
Skin, not otherwise specified	0.65 (0.47–0.89)	0.008
T‐stage, *n* (%)		
T0	1.00 (reference)	
T1	1.29 (0.92–1.83)	0.14
T2	1.47 (1.03–2.09)	0.03
T3	1.92 (1.28–2.85)	0.001
T4	1.90 (1.17–3.09)	0.009
Unknown T‐stage	1.07 (0.78–1.47)	0.66
Region, *n* (%)		
Northeast	1.00 (reference)	
South	0.81 (0.67–0.98)	0.03
Midwest	0.92 (0.76–1.13)	0.45
West	0.87 (0.69–1.09)	0.22
Unknown	1.50 (0.35–6.38)	0.58
Urban designation, *n* (%)		
Metropolitan	1.00 (reference)	
Urban	1.13 (0.93–1.37)	0.22
Rural	1.28 (0.77–2.12)	0.34
Unknown	0.99 (0.71–1.38)	0.96
Facility academic status, *n* (%)		
Nonacademic	1.00 (reference)	
Academic	0.84 (0.73–0.97)	0.02

The 5‐year OS of patients who received SNE was worse than patients with one positive node on TLND but similar to patients who received TLND with either two or three and more positive nodes (SNE 43.9% [95% CI 37.2–50.4]; TLND with one positive node 56.9% [95% CI 51.5–61.9]; TLND with two positive nodes 46.9% [95% CI 39.9–53.5]; TLND with three or more positive nodes 36.9% [95% CI 32.9–40.7]) (Figure 1b).

**Figure 2:**
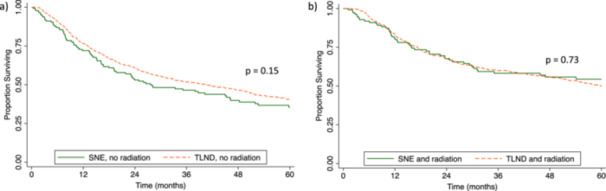
Kaplan–Meier curves of Merkel cell carcinoma patients with clinical nodal disease who underwent (a) single node excision (SNE) versus therapeutic lymph node dissection (TLND) with nodal basin radiation, and (b) SNE or TLND without nodal basin radiation.

In subgroup analyses by nodal basin radiation, no survival difference was observed between patients who underwent TLND with radiation or SNE with radiation (TLND 49.9% [95% CI 45.5–54.1] vs. SNE 54.4% [95% CI 44.1–63.6]) (Figure 2a). Equivalent survival outcomes were also seen for both surgeries without radiation (TLND 40.4% [95% CI 36.5–44.2], SNE 35.5% [95% CI 26.9–44]) (Figure 2b). In the cohort of patients who received an SNE, those who also received nodal basin radiation had an improved 5‐year OS compared to those who did not (SNE with radiation 54.4% versus SNE without radiation 35.4%, *p* = 0.006). With the receipt of radiation, the 5‐year OS of patients who received SNE was similar to patients undergoing TLND with one positive node (54.4% vs. 56.9%, *p* = 0.08).

## 4 Discussion

While practice guidelines have historically favored TLND for the treatment of regional lymph node metastases of skin cancers, there is a trend towards de‐escalation of nodal surgery. The present retrospective study found that after accounting for patient and tumor factors, SNE was not associated with a difference in OS and may merit further study as a safe alternative to TLND in MCC patients with clinical nodal disease. These results parallel those of a previous study done regarding the management of clinically positive nodes in patients with melanoma [6].

These results can have important implications for patients. Lymph node dissections are associated with seroma, infection, and lymphedema in up to 50% of cases [9]. In the MSLT‐II trial, patients who underwent observation after SLNB developed lymphedema at only 6% compared to the 24% of patients who underwent a CLND [5], supporting the notion that a more limited lymphadenectomy such as SNE could also potentially decrease these complications. Avoiding these complications may also allow for the more timely administration of adjuvant radiation therapy or not prevent a patient from the ability to receive adjuvant radiation therapy altogether. The results of our study suggest that a less extensive nodal surgery with likely concomitant decreased morbidity compared to a complete lymphadenectomy is not associated with worse survival outcomes. A less extensive dissection should be investigated further prospectively as a feasible option for management of a nodal basin with limited clinical disease, particularly, in the context of the radiosensitivity seen in MCC and with the advent of effective immunotherapies for MCC recurrence [10].

Consistent with previous studies [11], adjuvant radiation to the nodal basin was associated with an improved OS in patients undergoing SNE, likely due to decreased recurrence rates. A previous study demonstrated increased use of radiation therapy alone for the management of nodal micrometastases in MCC in the years following the MSLT‐II trial with similar survival outcomes [12]. Our results indicate that adjuvant radiation should be strongly considered in eligible patients although we are unable to describe the doses or fractionation of radiation used in this study as the NCDB reports that data per phase and we are unable to determine the dose delivered to a particular site (as in nodal basin vs. primary tumors). While survival outcomes for SNE appear inferior to those of TLND with a single pathologic node identified (likely secondary to stage migration from differential lymphadenectomy), this survival difference appears to disappear with the addition of adjuvant radiation.

The current study has several limitations. In the staging of MCC, cN1b disease encompasses a heterogeneous patient population with a variable number of clinically positive nodes. This heterogeneity in patient population is supported by the survival outcomes in patients undergoing SNE appearing to be an “average” of patients undergoing TLND stratified by nodal positivity. Along with being a retrospective study, this introduces potential selection bias for treatment with clinicians likely opting for SNE in patients with only one or few clinically positive nodes and TLND for patients with greater nodal burden of disease such as clinically matted nodes or involvement of adjacent nodal basins. There is also selection bias for treatment type with other both measured (i.e., age, higher T‐stage, and site of primary tumor) and unmeasured variables. With these limitations, these data must be cautiously interpreted, particularly, for patients who present with larger primary tumors and/or extensive nodal disease. However, the large sample size and similarity of outcomes provides important insight into the possibility of de‐escalating nodal surgery for those who present with limited disease and should be studied further. We are also unable to evaluate locoregional recurrence or disease‐specific survival as the NCDB does not capture those important oncologic outcomes. Since patients with MCC tend to be older and with increased comorbidities, OS may not accurately correlate with disease‐specific survival although the lack of association of extent of nodal surgery (SNE vs TLND) and OS persisted even when adjusting for factors such as age and co‐morbidities. The NCDB also does not collect information on postoperative complications or treatment toxicity, readmissions, or subsequent treatments upon relapse (either regional or systemic) which may be important when evaluating the risk/benefit ratio of either SNE or TLND in clinical practice.

For patients with clinical N1b MCC, TLND was not associated with a survival advantage compared to SNE. This suggests that SNE or a less extensive lymphadenectomy should be studied further as a feasible surgical option for patients with limited nodal disease, particularly, given the radiosensitivity of MCC and its responsiveness to novel immunotherapy regimens. Further studies with more granular data from multi‐institutional patient cohorts are needed to better compare complication rates, particularly, lymphedema, locoregional control, and disease‐specific survival between SNE and TLND, and allow for personalization of treatment to individual patients.

## Conflicts of Interest

The authors declare no conflicts of interest.

## Synopsis

For Merkel cell carcinoma patients with clinical nodal disease, therapeutic lymph node dissection is not associated with a survival advantage over single node excision. Single node excision combined with nodal basin radiation should be further studied as an option for management of the nodal basin.

## Data Availability

The data is subject to third party restrictions. The National Cancer Database (NCDB) is a joint project of the Commission on Cancer (CoC) of the American College of Surgeons and the American Cancer Society. The CoC's NCDB and the hospitals participating in the CoC's NCDB are the source of the deidentified data used herein. They have not verified and are not responsible for the statistical validity of the data analysis or the conclusions derived by the authors. Data are available at https://urldefense.com/v3/__https://www.facs.org/quality-programs/cancer-programs/national-cancer-database/puf/__;!!N11eV2iwtfs!tIPr1rWO6_pbdXygR2x5TKhlbx9I0ogcn51IMYVJ56CFRl6AsVAxjx7OJRJjFtooGF8mKsxNJyopqUi_qqlOmOc$ through an application process to investigators associated with CoC‐accredited cancer programs.
